# Hypothesis for heritable, anti-viral immunity in crustaceans and insects

**DOI:** 10.1186/1745-6150-4-32

**Published:** 2009-09-02

**Authors:** Timothy W Flegel

**Affiliations:** 1 Center of Excellence for Shrimp Molecular Biology and Biotechnology (Centex Shrimp); 2 Dept. of Biotechnology, Faculty of Science, Mahidol University, Rama 6 Road, Bangkok 10400, Thailand; 3 National Center for Genetic Engineering and Biotechnology (BIOTEC), Klong Luang, Pathumthani, 12120, Thailand; 4 National Science and Technology Development Agency (NSTDA), Klong Luang, Pathumthani, 12120, Thailand

## Abstract

**Background:**

It is known that crustaceans and insects can persistently carry one or more viral pathogens at low levels, without signs of disease. They may transmit them to their offspring or to naïve individuals, often with lethal consequences. The underlying molecular mechanisms have not been elucidated, but the process has been called viral accommodation. Since tolerance to one virus does not confer tolerance to another, tolerance is pathogen-specific, so the requirement for a specific pathogen response mechanism (memory) was included in the original viral accommodation concept. Later, it was hypothesized that specific responses were based on the presence of viruses in persistent infections. However, recent developments suggest that specific responses may be based on viral sequences inserted into the host genome.

**Presentation of the hypothesis:**

Non-retroviral fragments of both RNA and DNA viruses have been found in insect and crustacean genomes. In addition, reverse-transcriptase (RT) and integrase (IN) sequences are also common in their genomes. It is hypothesized that shrimp and other arthropods use these RT to recognize "foreign" mRNA of both RNA and DNA viruses and use the integrases (IN) to randomly insert short cDNA sequences into their genomes. By chance, some of these sequences result in production of immunospecific RNA (imRNA) capable of stimulating RNAi that suppresses viral propagation. Individuals with protective inserts would pass these on to the next generation, together with similar protective inserts for other viruses that could be amalgamated rapidly in individual offspring by random assortment of chromosomes. The most successful individuals would be environmentally selected from billions of offspring.

**Conclusion:**

This hypothesis for immunity based on an imRNA generation mechanism fits with the general principle of invertebrate immunity based on a non-host, "pattern recognition" process. If proven correct, understanding the process would allow directed preparation of vaccines for selection of crustacean and insect lines applicable in commercial production species (e.g., shrimp and bees) or in control of insect-borne diseases. Arising from a natural host mechanism, the resulting animals would not be artificially, genetically modified (GMO).

**Reviewers:**

This article was reviewed by Akria Shibuya, Eugene V. Koonin and L. Aravind.

## Background

Pathologists of shrimp and insects are presented with the paradoxical situation that individual viral pathogens may sometimes cause severe disease but may also be carried as active infections for long periods (including a full lifetime) without measurable signs of disease (see [1,2]for reviews). In addition, it is curious that the massive viral disease outbreaks experienced in shrimp cultivation ponds have not, so far, been reported from natural populations in areas where severe pond outbreaks have occurred [3]. This is despite the common detection of the relevant viruses in grossly normal individuals from wild shrimp populations (e.g., [4]).

Since shrimp and insects are able to survive for long periods with persistent infections, it is important in viral challenge tests to determine whether grossly normal test animals are free of various viral pathogens prior to challenge and whether survivors are infected with the challenge virus post-challenge. After a challenge test, the individuals may be "uninfected", "infected but not diseased" or "infected and diseased". Individuals in the "infected and diseased" group may or may not recover. If they do recover, they could conceivably end up either "uninfected" or "infected but not diseased". The latter would depend upon whether they could clear the pathogen or whether they remained persistently infected. These different outcomes may vary with such things as viral isolate type and shrimp target species or shrimp life stage within a single species [5,6]. Although distinguishing between "uninfected" and "infected but not diseased" survivors has important ramifications for interpretation of shrimp and insect responses to viral challenges, it is unfortunate that survivors are sometimes ambiguously labelled simply as "resistant".

The capacity of shrimp and other arthropods to carry single or multiple viral infections without gross signs of disease is evident even with insect cells where persistent single infections or co-infections can be easily produced [7]. A theoretical viral accommodation model was proposed to account for the ubiquity of persistent infections without signs of disease and directions for testing the model were also proposed [1,2]. Briefly, the updated model [2] proposed that shrimp and other arthropods have an active (adaptive) mechanism for accommodation of viral pathogens in a manner a) that leads to persistent infections without signs of disease, b) that specifically blocks viral-triggered, massive apoptosis called kakoapotosis (Greek kako = bad or detrimental) and c) that also provides some protection against mortality upon subsequent superinfection with the same and possibly other viruses [8,9]. An essential element for the viral accommodation model was an initially unknown pathogen recognition mechanism or memory mechanism [1] that allowed the host to respond specifically to each viral pathogen or variant. The recognition mechanism was later proposed to be based on the presence of the viruses themselves in persistent infections [2]. However, recent information suggests that the specific response mechanism may be based on heritable, viral genome fragments inserted into shrimp and insect genomes.

## Presentation of the hypothesis

### Non-retroviral sequences in arthropod genomes

In 2006, Tang and Lightner [10] reported the occurrence of non-infectious sequences of Penaeus stylirostris densovirus (*Pst*DNV) (formerly called infectious hypodermal and hematopoietic necrosis virus or IHHNV) inserted into the chromosomal DNA of Penaeus mondon from East Africa and Australia. This necessitated a change in the routine method for detection of the infectious form of the virus [11]. WSSV-like sequences have also been reported from the genome of P. monodon from Australia [12]. In addition, many viral-like sequences have been found in a Fosmid clone library constructed from the P. monodon genome [13]. A similar phenomenon has been reported from insects [14,15]. Curiously, many of these reported viral inserts are not retroviral in origin, and it is not known how they became inserted into host genomes. On the other hand, the *Pst*DNV fragments in shrimp were reported to be associated with transposable elements [10]. This raised the question as to whether shrimp and other arthropods have a mechanism for integration of viral sequences into their genomes in a manner that may play some role in subsequent occurrence of persistent infections without signs of disease. For example, *Pst*DNV is endemic in the range of P. monodon, with which it generally causes no disease [16,17]. It was not discovered until it jumped from grossly normal stocks of *P. monodon *moved to the Americas for cultivation near the native species *P. stylirostris *with which it caused massive mortality [18].

### Host reverse transcriptases (RT) and integrases (IN)

If general viral sequences inserted into the host genome constitute a potential mechanism for a specific, heritable immune response in shrimp and insects, then there should be a natural process that allows for insertion of non-retroviral sequences into their genomes. A search of a shrimp EST database revealed 5 different RT tags and 7 different IN and transposase tags [19]. RT and IN from retrotransposons, "endogenous retroviruses", retrovirus-like elements with long terminal repeats (LTR) or other viral-like elements in the genome are well known in the insect literature [20-22]. Some of these are referred to as insect erantiviruses, metaviruses or pseudoviruses [20,23-25]. The widespread occurrence of these virus-like elements in arthropod genomes suggests that they may play some functional role in their hosts, especially for those types that are transmitted via the genome only.

### The hypothesis

It is possible that random integration of viral genome fragments into the host genome by host-derived RT and IN could lead to antisense mRNA transcripts capable of suppressing propagation of the same virus. Since the process would give rise to RNA via specific molecular steps, it could be called an immunospecific RNA (imRNA) generation mechanism. Antisense constructs of Taura syndrome virus (TSV) have been shown to be effective in protecting against TSV in transgenic shrimp [26]. Similar results have been obtained using viral antisense sequences in transgenic plants [27-30]. These antisense sequences provide protection via the RNA interference (RNAi) pathway that has been verified in both shrimp [31,32] and insects [33,34]. If a natural process for imRNA generation does exist, it would constitute a new type of heritable, adaptive response to viral pathogens in crustaceans and insects, and perhaps other arthropods.

A good target for this imRNA generation process would be pathogen mRNA, since it constitutes a common metabolite for both RNA and DNA viruses. It would also allow for molecular mechanisms to distinguish between host and pathogen mRNA. For example, mRNA of cytoplasmic origin might be a key target. The variety of RT-like sequences in the shrimp and arthropod databases supports the possibility that they could be involved in a non-self, "pattern recognition" processes similar to those pathogen-associated molecular patterns (PAMP) characteristic of other innate immune responses of invetebrates [35-37]. However, in contrast to other PAMP-based innate immune responses, the imRNA generation process would have to be considered a specific and heritable, adaptive response.

In summary, it is hypothesized that viral mRNA in shrimp and insects is recognized as foreign (possibly by host RT) and processed by host RT and IN for insertion of random cDNA fragments into host genomic DNA. Some of these fragments generate antisense, imRNA sequences that bind with viral mRNA to induce the host RNAi mechanism and suppress viral propagation. This leads to low-level, active infections where the host exhibits no signs of disease but may remain infected and infectious for naïve hosts. During reproduction, random assortment of chromosomes allows for rapid mixing of protective inserts for various pathogens, and selection from copious offspring allows for rapid development of a population with multiple virus tolerance. For example, the black tiger shrimp *P. monodon *has 39 pairs of chromosomes leading to approximately 450 billion possible chromosome combinations from females that produce in the order of 1 million eggs per spawn in multiple spawns. By comparison, the number of possible combinations from the 23 pairs of human chromosomes is only in the order of 8 million. For insects where recombination by crossover would be required because of small chromosome numbers (e.g., *Drosophila*), there may be compensation in the form of extremely large populations, very short generation intervals and high numbers of offspring.

## Testing the hypothesis

Many experiments can be carried out to test this hypothesis. However, some relatively simple, preliminary tests would reveal whether further pursuit would be fruitful or not. For example, it predicts that host RT will be capable of using viral mRNA sequences as a substrate for generation of cDNA sequences and that these will be inserted into the host genome. Therefore, viral challenge followed by genomic analysis of many infected, long-term survivors (e.g., [38]) should reveal the presence of viral genome fragments present in the host genome. These inserts should be different for different individuals. Good candidate viruses would be RNA viruses (not retroviruses) since it would be easy to prepare total host DNA, treat it with RNase and probe it by dot blot for positive hybridization with the total, labelled viral cDNA. Positive samples could be tested further with labelled viral sub-clones to determine what fragments were inserted. Further analysis would reveal the locations, polarity and potential imRNA productivity of the inserts. Natural arthropods with viral tolerance, or arthropods genetically selected for this character would also be good targets for this type of research. For example, shrimp genetically selected for high tolerance to the positive-sense ssRNA virus TSV, are widely used in the shrimp industry and are easily available [39,40]. Also, the genomes of natural *P. monodon *specimens should contain inserts of *Pst*DNV additional to those already reported [10]. For these insert-screening assays, it would be necessary to include DNA extracts from eggs and sperm that have been separated from supporting tissues, since inserts in the germ cells would be required for heritable immunity.

According to the hypothesis, the host RNAi system should be critical to the maintenance of low-level persistent infections in animals that survive viral challenges. Thus, knock-down of key gene transcripts such as those critical to the RNA-induced silencing complex (RISC) should lead to an increase in viral replication and perhaps mortality in such individuals. Targeting RT and IN knockdown may be less informative if the host has more than one form of each.

## Implications of the hypothesis

The hypothesized process for random imRNA production is somewhat analogous to that for random antibody production in vertebrates where many antibodies are produced but only some are protective. By understanding the process, it became possible to select only protective antigens for use in vaccination programs. Similarly, preparation of appropriate viral constructs or mimics could lead to insertion of protective antisense fragments into shrimp and crustacean genomes by a natural host process that could not be called artificial genetic modification. Thus, the resulting animals could not be considered genetically modified organisms (GMO) and could be used in breeding programs to rapidly develop disease tolerant stocks for animal production (e.g., shrimp and bees) or special stocks to control disease transmission by insect vectors of viral diseases such as Dengue fever. Similar tests could be carried out in other arthropod groups to determine whether the process occurs more widely in the phylum.

## Conclusion

It is hypothesized that crustaceans and insects recognize viral mRNA and use it as a target for random insertion of viral sequences into their genomic DNA using endogenous RT and IN. These inserted sequences can produce immuospecific RNA (imRNA) that binds with viral mRNA to induce the RNAi mechanism and suppress viral propagation (Appendix). The hypothesis fits with the general principle of invertebrate immunity based on a non-host "pattern recognition" process. If correct, understanding the process would allow directed preparation of vaccines for selection of crustacean and insect lines applicable in commercial production (e.g., shrimp and bees) or in control of insect-borne diseases. Arising from a natural host mechanism, the resulting animals could not be considered artificially, genetically modified organisms (GMO).

## Abbreviations

RT: reverse transcriptase(s); IN: integrase(s); imRNA: immunospecific RNA; RNAi: RNA interference; GMO: genetically modified organism(s); PstDNV: Penaeus stylirostris densovirus of shrimp; IHHNV: infectious hypodermal and hematopoietic necrosis virus of shrimp; WSSV: white spot syndrome virus of shrimp; LTR: retrovirus-like elements with long terminal repeats; TSV: Taura syndrome virus of shrimp.

## Competing interests

The author declares that they have no competing interests.

## Appendix

### Summary of the steps in the imRNA response mechanism

1. Infection of host cells by an RNA or DNA virus

2. Generation of viral mRNA

3. Recognition of foreign viral mRNA possibly by host reverse transcriptases (RT)

4. Random synthesis of cDNA fragments from foreign mRNA by host RT

5. Transport of the random cDNA fragments into the nucleus

6. Integration of the cDNA fragments into the host genome via host integrases (IN)

7. Production of viral antisense, immunospecific RNA (imRNA) by some inserts

8. Formation of dsRNA by imRNA binding with viral mRNA

8. Induction of the host RNA interference (RNAi) mechanism

9. Reduction in viral load, leading to persistent viral infections.

## Reviewer's comments

### Reviewer's report 1

Akira Shibuya (Department of Immunology, Institute of Basic Medical Sciences, University of Tsukuba, Tsukuba Science-City, Japan)

#### Reviewer Comments

The author describes a hypothesis that explains how arthropods can persistently carry one or more viral pathogens without signs of disease. His conclusion is that host endogeneous RT and IN recognize viral mRNA and insert virus-derived cDNA sequences into the host genome, producing imRNA that suppresses viral propagation. This scenario is very interesting and persuasive, because several previous reports may support the idea. However, I have several concerns, as follows:

To inherit the resistance to a virus in the next generation, it is required that the viral-derived DNA sequence be inserted into host germ cells. However, this is not clearly described and discussed.

#### Author's response

This is a very important point that was implicit in the hypothesis but not specifically stated. It is now included in the section on testing the hypothesis. As far as I know, no tests have been done specifically on the presence of such viral inserts in host germ cells.

#### Reviewer Comments

In my opinion, the term of "memory" in the title is not suitable, if this is truly inherited, because memory itself should be acquired and continue within one generation. If a viral sequence is inserted into germ cells, this is actually inherited and programmed, but not acquired by the next generation.

#### Author's response

I think this point is debatable, since DNA is a memory molecule. The hypothesis proposes that the ability of arthropods to specifically respond to viruses and their variants requires an initial infection event followed by a process that leads to genome insertions and specific RNAi responses. I believe that the insertion could be considered a kind of memory that may be passed on via germ cells to the next generation that would be capable of the same, specific RNAi response. On the other hand, it is possible that some new technical term may be required to set this phenomenon apart from the type of acquired immunity related to antibodies. To avoid the issue somewhat in this paper, I have reduced use of the word "memory" in the text and replaced it with phrases such as a heritable and specific pathogen response mechanism.

#### Reviewer Comments

An abbreviation list may be useful for readers.

#### Author's response

An abbreviation list has been added.

### Reviewer's report 2

Eugene V. Koonin (NCBI, NLM, NIH, Bethesda, MD, USA).

#### Reviewer Comments

A very interesting and, in my opinion, promising hypothesis on the mechanism of heritable resistance to pathogens in arthropods. The idea is that short regions of viral mRNAs are copied by host reverse transcriptase and the copies are integrated in the genome where a few can be utilized to produce small RNAs interfering with the respective virus (imRNAs). The hypothesis is compatible with the findings of numerous virus-specific inserts in arthropod genomes and appears quite plausible.

Moreover, the idea seems particularly interesting in light of the rapidly accumulating similar findings in plants [41] and especially the discovery of the elaborate system of defense against mobile genetic elements in bacteria and archaea that functions on the same principle [42, 43, 44]. It is an intriguing possibility that defense system that involve integration of foreign DNA into genomes and thus represent a form of Lamarckian inheritance function in most cellular life forms.

#### Author's response

I agree with these comments.

### Reviewer's report 3

L. Aravind (NCBI/NLM/NIH, Bethesda MD, USA)

#### Reviewer Comments

It this article, TW Flegel presents a hypothesis for the phenomenon of specific memory in viral accommodation in arthropods. This is postulated as being behind the low level persistence of viruses without noticeable deleterious effects on the host but instead conferring resistance to super-infection. The hypothesis for memory presented here tries to exploit two ideas: 1) Integration of DNA copies of the viral genome or fragments thereof into the host genome. 2) A post transcriptional gene silencing process utilizing transcripts derived from RNAs from these integrated copies. The hypothesis as presented here is certainly plausible as it invokes two processes that are known to occur in eukaryotic genomes. Further, there is already evidence for small RNA dependent defensive mechanisms against genomic parasites, such as the piwiRNAs. The mechanism presented here is not very different in a general sense from this and more well know RNAi based defenses against RNA viruses.

My primary criticism regarding this work is the evidence the author supplies from existing arthropod genome sequences for supporting the hypothesis. While the author uses shrimp as the model, the statements in the article clearly imply certain generality across arthropods. Given that in phylogenetic terms the insects are nested inside the crustacean radiation extending this generalization, at least to insects, is not without foundation. However, more clarification would be useful as to whether the author implies that this memory phenomenon would be restricted to crustaceans (including insects) or other all arthropods or more generally across animals or even eukaryotes.

#### Author's response

The hypothesis currently applies to crustaceans and insects only because my evidence about non-retroviral inserts comes exclusively from these groups. However, the possibility that the mechanism may be more widespread to include all arthropods and perhaps other eukaryotes may be raised. I have changed the text and the title of the manuscript to make it clear that I refer specifically to crustaceans and insects.

#### Reviewer Comments

Further, this generalization technically opens up the scope for detection of viral sequences such as those postulated by the author in the several completely sequenced and publicly available genomic sequences of insects and crustaceans. However, while the author cites earlier papers for integration of flaviviruses and certain prawn viruses, no direct evidence from published genomic sequences is offered. Such an analysis would have made the case much stronger.

#### Author's response

I did a very preliminary search of complete insect genomes using a few complete insect virus sequences but did not find strong evidence to support my hypothesis. For example, I used the complete sequence of Aedes albopictus densovirus (NC_004285) for a discontinuous Blastn search against *Aedes aegypti*. I found only small fragments of the densovirus sequence of around 20-40 base pairs that matched mosquito sequences with identities of 90-100%. Curiously, many of these were associated with repeat regions including mobile, transposable elements like Ty1_copia, Ty3_gypsy, tRNA-related SINEs, m8bp MITEs, PIF transposons, pogo transposons, Jockey Non-LTR, Pao_Bel LTR, R1 Non-LTR, etc. Matches to similar small fragments were obtained using the same virus in a search against the human genome, but my quick perusal did not reveal that these were related to repeat regions or mobile, transposable elements. I was hesitant to make any conclusion from this very superficial examination. I believe that more detailed work would be required using preliminary, pre-assembly sequences that were employed to arrive at the polished, complete insect genome sequences. Even then, the hypothesis proposes that different individual crustaceans or insects will be likely to carry different combinations of inserts, possibly in different locations in the genome and probably all in "non-functional" regions such as those occupied by transposable elements and multiple repeats. Thus, use of complete genome data arising from a very small number of standardized DNA samples (probably derived from laboratory strains) might also affect the final result. Thus, I suggest that this type of analysis should be included as another test of the hypothesis.

#### Reviewer Comments

Secondly, if this mechanism is indeed widespread across arthropods the question arises as to whether a dedicated integrase and RT systems has been set aside for this. The author mentions the endogenous retroviruses that might provide integrases and RTs, but there is no more direct evidence presented for their involvement in cycling viral cDNAs/DNAs into the genome. In mammals there are some retrovirus derivatives with specialized NYN RNAse domains and integrases (such as those we reported sometime back, see PMID: 17114934) [45] that could indeed function in a similar process of anti-viral defense. Detection of such components in insect/crustacean genomes could further strengthen the case and even provide targets for directed experimental studies.

#### Author's response

As with my response to the question on whole genome searches, these are good questions that can be added to the list of tests of the hypothesis. The evidence shows that non-retroviral inserts can be found in insect and crustacean genomes. The hypothesis proposes that they arise from the activity of host RT and IN that work together in a directed process that results in an immunological response. Any host RT or IN would be a potential candidate as a player in this process.

#### Reviewer Comments

A figure summarizing the processes in the hypothesis would have been useful.

#### Author's response

A a figure of the stepwise process has been added to the manuscript

#### Reviewer Comments

In terms of testing the hypothesis: Could insect models such as *Apis*, *Tribolium *or *Drosophila *with already sequenced genomes be used? There is already some understanding of the RNA based interference in them.

#### Author's response

Yes, existing insects with complete genomes would be excellent models to test the hypothesis. Please see my response above to the previous question on analysis of complete genome sequences.

#### Reviewer Comments

Could the mechanism involve any kind of link to the ubiquitin-based viral protein degradation system? This angle is suggested by expansions of F-box and other Ub-E3 ligase components in various viruses.

#### Author's response

Although this may be possible, it is not critical to the hypothesis. It could be another avenue of research.

#### Reviewer Comments

In discussion of retroviruses like elements in arthropod genomes the author mentions "pseudoviruses, metaviruses etc" and provides citations such as ref [25]. The elements presented here are not necessarily retroviruses; in fact they are mobile elements that resemble DNA viruses such as adenoviruses and NCLDVs in certain ways. For example we note such elements in PMID: 15466593 [46] (this describes the same elements mentioned in ref 25 of this publication well before that publication) and PMID: 18753784 [47]. It would be worthwhile if the author more clearly distinguishes the other types of virus-like elements which are retrovirus-like.

#### Author's response

Due to the general nature of the hypothesis, I tried to use review papers of various retrovirus-like elements or other viral-like elements that might provide RT and IN that could participate in the process of viral mRNA recognition and directed insertion of cDNA fragments into the host genome. I have modified the text to clarify my intended meaning.
