# Hypothesis for heritable, anti-viral immunity in crustaceans and insects

**DOI:** 10.1186/1745-6150-4-36

**Published:** 2009-10-01

**Authors:** Timothy W Flegel

**Affiliations:** 1 Center of Excellence for Shrimp Molecular Biology and Biotechnology (Centex Shrimp; 2 Dept. of Biotechnology, Faculty of Science, Mahidol University, Rama 6 Road, Bangkok 10400, Thailand; 3 National Center for Genetic Engineering and Biotechnology (BIOTEC), Klong Luang, Pathumthani, 12120, Thailand; 4 National Science and Technology Development Agency (NSTDA), Klong Luang, Pathumthani, 12120, Thailand

## Abstract

Correction to Flegel, TW: Hypothesis for heritable, anti-viral immunity in crustaceans and insects. *Biology Direct *2009, 4:32.

## Correction

The published version of this article [1] omits the references from the Reviewer's Comments section (citations 41 to 47). The missing references appear here in their respective order [2-8].
